# Lipid incorporated biopolymer based edible films and coatings in food packaging: A review

**DOI:** 10.1016/j.crfs.2024.100720

**Published:** 2024-03-15

**Authors:** L. Susmita Devi, Amit K. Jaiswal, Swarna Jaiswal

**Affiliations:** a Department of Food Engineering and Technology, Central Institute of Technology Kokrajhar, Kokrajhar, BTR, Assam, 783370, India; b Sustainable Packaging & Bioproducts Research (SPBR) Group, School of Food Science and Environmental Health, College of Sciences and Health, Technological University Dublin - City Campus, Central Quad, Grangegorman, Dublin, D07 ADY7, Ireland; c Sustainability and Health Research Hub, Technological University Dublin, City Campus, Grangegorman, Dublin, D07 H6K8, Ireland

**Keywords:** Fruits and vegetables, Postharvest shelf life, Food packaging and preservation, Edible films and coatings, Hydrophobicity, Water vapor permeability

## Abstract

In the evolving landscape of food packaging, lipid-based edible films and coatings are emerging as a sustainable and effective solution for enhancing food quality and prolonging shelf life. This critical review aims to offer a comprehensive overview of the functional properties, roles, and fabrication techniques associated with lipid-based materials in food packaging. It explores the unique advantages of lipids, including waxes, resins, and fatty acids, in providing effective water vapor, gas, and microbial barriers. When integrated with other biopolymers, such as proteins and polysaccharides, lipid-based composite films demonstrate superior thermal, mechanical, and barrier properties. The review also covers the application of these innovative coatings in preserving a wide range of fruits and vegetables, highlighting their role in reducing moisture loss, controlling respiration rates, and maintaining firmness. Furthermore, the safety aspects of lipid-based coatings are discussed to address consumer and regulatory concerns.

## Highlights

• Lipid-based films/coatings offer a sustainable solution to enhance food quality and shelf life.
• Coatings integrate lipids with biopolymers, improving thermal, mechanical, and barrier properties.
• Waxes such as carnauba and beeswax significantly improve water resistance in fruits.
• Effectively preserve fruits & veg. by reducing moisture loss and controlling respiration rates.
• Review shows a gap in studies on lipid-biopolymer films, suggesting need for more research.

## Graphical abstract

**Figure:**
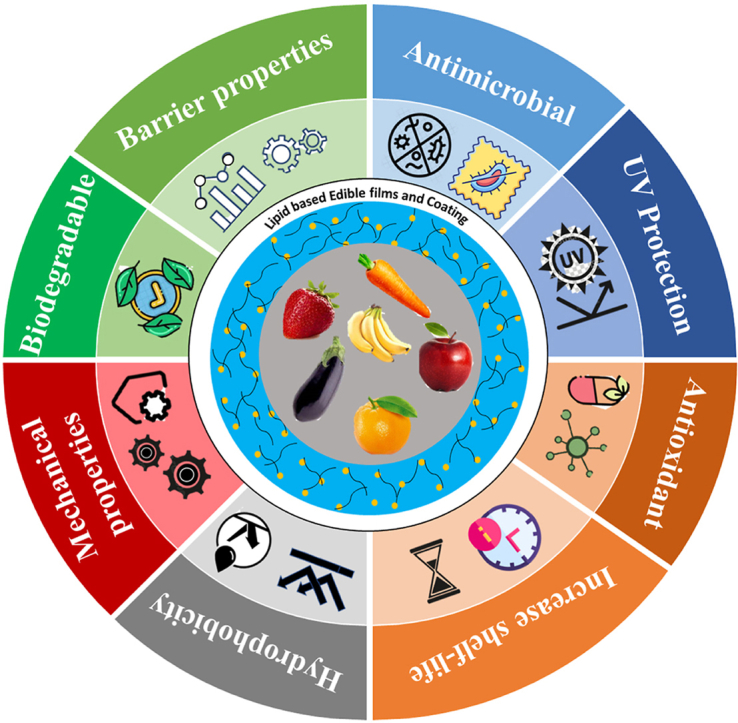

## 1 Introduction

Food packaging plays a vital role in the food supply chain, protecting food from harmful environmental impacts and preserving its structure for a longer period, thus maintaining its nutritional and flavour qualities (Trajkovska Petkoska, Daniloski, D'Cunha, Naumovski and Broach, 2021). Over the years, traditional commercial food packaging materials such as polypropylene, polystyrene, and polyethylene have been used to protect food against environmental changes and microbial contamination (Zhang et al., 2020). These traditional petroleum-based plastic food packaging materials pose significant environmental challenges due to their non-degradable nature, leading to ecological issues like threats to aquatic life and air quality degradation (Shaikh et al., 2021). The food packaging industry is currently concerned about food safety and environmental protection. Therefore, the food industry and scientists are investigating various approaches for a better alternative that meets environmental standards, as well as using biodegradable ingredients in food packaging (Tahir et al., 2019).

Biodegradable packaging made from natural polymers appear to be promising alternative to plastic packaging. These materials are utilized in the production of edible films and coatings, forming a protective layer around the food surface to protect against various factors (Mohamed et al., 2020). In the food packaging industry, edible films and coatings are of great interest due to their edible properties, which can reduce packaging waste as well as exhibit impressive resistance to moisture, aroma transfer, solvent transfer, and oxidation (Atta et al., 2022). They further enhance food safety and physical strength, reduce particle clustering, and prevent microbial growth, thereby extending the shelf life of food (Milani and Nemati, 2022). Edible films are preformed structures used to wrap around a food product to protect it from degradation. Additionally, they serve as carriers for active substances such as flavours, antimicrobials, and antioxidants. Whereas the edible coatings are typically applied directly to the surface of the food by either spraying or dipping in liquid form, resulting in the formation of micro-layers of film on the food surface, or through electro-spraying, which produces a thin and uniform coating (Hassan et al., 2018).

Films and coatings are generally prepared from biopolymers such as polysaccharides, proteins, and lipids (Mohamed et al., 2020). Each group of materials has some benefits and drawbacks. In general, protein and polysaccharide films have strong mechanical characteristics, a strong barrier against oxygen at intermediate and low relative humidity, but a weak barrier against water vapor due to their hydrophilic nature (Mohamed et al., 2020; Rhim and Shellhammer, 2005). Whereas lipid films provide hydrophobicity and cohesion, lipid coatings provide excellent barriers to moisture and oxygen, ensuring long-term food quality (Rhim and Shellhammer, 2005). Packaging made from a single material often doesn't meet the requirements for ideal packaging due to inherent limitations. Therefore, forming composites is a strategy to improve the characteristics of food packaging. Composite films and coatings can be formed through the combination of different packaging materials. However, films and coatings made with hydrocolloids and lipids have greater effectiveness than composite films made from blends of similar types of materials (Yousuf et al., 2022). Lipid materials have different characteristics that determine their effectiveness in composite films and coatings, including their structure, physical state (liquid or solid), chemical arrangement, hydrophobicity, and interactions with other components (Pérez-Gago and Rhim, 2013; Rhim and Shellhammer, 2005).

Waxes, fatty acids, resins, and neutral lipids are the most commonly used lipid compounds for the fabrication of edible films and coatings (Pérez-Gago and Rhim, 2013). A liquid lipid offers less resistance to gas and vapor transmission than a solid lipid. Lipid-based edible films can also apply to cereals, chocolates, dairy products, confectionery products, frozen products, fish, poultry meat, and dried products (Milani and Nemati, 2022). Over the past years, numerous research studies have focused on edible films and coatings for food packaging, primarily highlighting single-component or composite formulations. However, there's been a lack of detailed review papers specifically examining lipid-incorporated biopolymer-based edible films and coatings in food packaging, with only a handful of related publications available, such as the works of Debeaufort and Voilley in the year 2009 (Debeaufort and Voilley, 2009), Rhim and Shellhammer in the year 2005 (Rhim and Shellhammer, 2005), Pérez-Gago and Rhim in the year 2014 (Pérez-Gago and Rhim, 2014), and Morillon and team in the year 2002 (Morillon et al., 2002), most of which are over a decade old. More recently, Milani & Nemati in 2022) (Milani and Nemati, 2022), Hassan and team in 2018) (Hassan et al., 2018), and Yousuf and team in 2021 (Yousuf et al., 2022) have contributed reviews on similar topics, but these still do not adequately cover the novelty and comprehensiveness of the subject matter. Consequently, there was a necessity for an updated review to address this gap. This critical review aims to offer a thorough overview of recent developments in lipid-incorporated biopolymer edible films and coatings, emphasizing the significance of lipids in food packaging. It delves into their functional properties, formulation techniques, film fabrication methods, and their application in preserving various fruits and vegetables. Furthermore, the review also touches upon safety considerations associated with lipid-incorporated films and coatings.

## 2 Lipids and their role in food packaging

Lipids are organic substances that originate from living things, including plants, animals, and insects. However, they are substances that are largely insoluble or hardly soluble in water but soluble in a few specific organic solvents like methanol, hexane, diethyl ether, benzene, and chloroform and require high temperatures to be processed (Akoh and Min, 2002). Lipids are grouped into three primary categories: simple, derived, and compound lipids. Simple lipids are further subcategorized into fats and oils, along with waxes. Compound lipids, on the other hand, are divided into two classes: phospholipids and glycolipids. The third category of lipids, derived lipids, is intricately classified into steroids, terpenes, and carotenoids. Among these, simple lipids, particularly fats and oils, play a prominent role in food and nutritional processes. Oils and fats, composed mainly of triglycerides, are widely prevalent lipids in various food sources, both from animals and plants (Zubair et al., 2021).

Lipid films and coatings have been protecting food for centuries to prevent moisture loss. It is believed that the first edible coating was used in China in the twelfth century on oranges and lemons (Andrade et al., 2012a, Andrade et al., 2012b). In the United States, the first edible coatings were used in the sixteenth century, when fresh produce was coated with lipid-based substances such as carnauba wax, paraffin wax, and emulsion oil-in-water to prevent moisture loss. Later, coatings were also applied to add shine to fresh produce in the twentieth century (Galus and Kadzińska, 2015). Pure lipid films and coatings are rarely used in practice because they are difficult to produce due to their mechanical weakness compared to hydrocolloids. Additionally, the properties of films and coatings, including barrier, mechanical, thermal, and optical characteristics, are significantly influenced by the formulation and preparation methods employed (Yousuf et al., 2022). Therefore, composite films and coatings are developed by incorporating hydrophobic lipid compounds (hydrophobic) such as oleic acid, beeswax, or glycerides with other hydrophilic biopolymers like proteins or polysaccharides to improve the film's functionality (Yousuf et al., 2022). The use of lipids in the food industry is currently gaining attention, with their unique properties such as providing hydrophobicity and acting as a barrier against water-vapor transfer. The mass transfer resistance exhibited by lipid compounds to gas and vapor transfer is primarily attributed to their hydrophobic nature and structural characteristics. Lipids, thus, emerge as cutting-edge sustainable and renewable raw ingredients, expanding their applications in food packaging with improved antioxidant and antibacterial properties in addition to other physiochemical qualities (Zubair et al., 2021).

Lipids, known for their economical and abundant presence in nature, are extensively used in edible films and coatings. In addition, lipids minimize moisture loss and packaging costs while imparting a glossy finish to food products, enhancing their visual appeal (Yousuf et al., 2022). Several studies have investigated the addition of lipids to the biopolymer film matrices to change various hydrocolloid film properties. A composite transglutaminase gelatin film containing beeswax exhibited higher moisture resistance and tensile strength compared to the film without a lipid component (Zheng et al., 2022). In another study, Liu et al. (2019) studied how glycerides affect the film-forming ability of maize starch and observed that starch films with glycerides exhibited increased tensile strength, decreased elongation at break, and improved resistance to water compared to films made from native starch. It is possible that the formation of maize starch-glyceride inclusion complexes is responsible for the increase in tensile strength (Liu et al., 2019). Recently, Butt et al. (2023) revealed that adding carnauba wax to films based on sodium alginate and whey protein-based films improved the moisture barrier characteristics (Butt et al., 2023). The development of these complexes contributes to enhanced strength in the polymer matrices.

### 2.1 Wax

Waxes are hydrophobic materials made up of long and medium-length carbon atom chains. A wax may take the form of synthetic or natural wax, depending on its source (Goslinska and Heinrich, 2019). Compared to other lipids, they have great hydrophobicity, making them superior water barriers. Their high hydrophobicity results from a high content of long-chain esters and alkanes, long-chain fatty acids, and alcohols (Nurul Syahida, Ismail-Fitry, Ainun and Nur Hanani, 2020). Various studies have examined the use of wax coatings to prevent the desiccation of various fruits and vegetables. For example, composite films or films with added wax, such as chitosan-beeswax edible coatings, improved the shelf life of sapodilla fruits by up to 17 days compared to uncoated fruits. Incorporating beeswax into chitosan coating formulations reduces microbial infection and weight loss while maintaining skin firmness and color (Pashova, 2023b). Similarly, applying carnauba wax nanoemulsion as an edible coating to fresh tomatoes prolonged shelf life by up to 15 days (Miranda et al., 2022). Additionally, integrating carnauba wax into arrowroot starch-based edible films through emulsion technology enhanced their hydrophobic characteristics, reduced moisture content, water vapor permeability (WVP), and thermal stability, and improved light barrier properties, exhibiting the best overall properties for food packaging (Oliveira Filho et al., 2020). Furthermore, candelilla wax and guar gum-based edible coatings also showed effective potential for prolonging blackberry shelf life by up to 6 days and also providing a barrier to reduce physiological weight loss and fruit decay, with no effect on firmness. whereas uncoated blackberries decayed within 4 days (Ascencio-Arteaga et al., 2022). These natural waxes are generally considered more acceptable in the food industry as they help reduce stickiness and enhance its surface appearance (Pashova, 2023a).

Many countries, including the United Kingdom, Norway, and Japan, have restricted or banned the addition of petroleum-based waxes, like polyethylene, in the food industry due to increasing consumer demand for environmentally friendly packaging as well as an increase in energy costs that raises the cost of conventional packaging materials (Rhim and Shellhammer, 2005). However, in oenology, wine bottle corks are often coated with paraffin wax or beeswax to protect the cork from sopping and provide wine with flavour. This coating method can be replaced instead of using plastic corks in wine bottles (Debeaufort and Voilley, 2009). Common packaging materials, such as paperboard and paper used in drinks and food packaging, are often coated with wax to increase the shelf life and water resistance of the food products. It is preferable to use natural waxes instead of synthetic waxes since they are derived from sustainable sources like plants or insects (Nurul Syahida et al., 2020).

### 2.2 Resins

Resins are non-volatile plant compounds that either naturally drip from the surface of a plant (surface resins) or can be acquired through cutting or infection (internal resins), except for lac, called shellac which is produced from the excretion of the female “lac bug” (Kerria lacca) on trees. All other natural resins are of plant origin and can be converted into a polymer (Karak, 2016). Resin is also insoluble in water and soluble in organic solvents. They are amorphous, stable, inert, and become sticky when heated also they are fusible and lack sharp melting points. Resins are represented by coumarone indene, wood rosin, and shellac and these are the primary coating elements (Galus and Kadzińska, 2015; Rhim and Shellhammer, 2005). Shellac resin consists of esters having hydroxyl and carboxylic groups. It possesses good water resistance and barrier-forming properties with excellent film-forming properties, which makes it a popular coating and functional material. Furthermore, being an animal-based resin, it has natural properties as well as good biodegradability and biocompatibility. The FDA has also approved its use in food and drug applications (Li et al., 2022). In the agro-food industries, shellac resin is widely utilized for preserving fruits and vegetables by preventing gas, water, and microbial spoilage. In the pharmaceutical sector, it has been applied to control drug delivery, enteric coating for drugs, and protection of moisture loss (Amin et al., 2021). To boost the mechanical and functional properties of shellac films, the particle size or crosslinking of the molecules in shellac molecule can be minimized (Prawatborisut et al., 2019).

Numerous studies on resin for packaging applications have explored its inclusion in the formulation of edible coatings. For example, shellac-based surface coating, when combined with passive modified atmosphere packaging, effectively prolonged the shelf life of fresh green chillies for up to 48 days, exhibiting lower respiration rates compared to the control (Chitravathi et al., 2016). In another study, A composite shellac-gelatin coating acts as an effective physical barrier and preserves the postharvest quality of bananas for over 30 days (Soradech, Nunthanid, Limmatvapirat, & Luangtana-anan, 2017). Khorram et al. (2017) have also reported that shellac coatings applied to 'Valencia’ oranges resulted in reduced weight and firmness loss, forming a non-sticky, odorless layer, and providing the highest fruit gloss during storage (Khorram et al., 2017). In recent years, gum rosin, a by-product of pine resin, has been highly valued and drawn attention in the food packaging industry as a natural cost-effective additive (i.e., compatibilizers, stabilizers, and/or plasticizers) adding intriguing properties such as improvement in the viscoelastic and tensile properties to the poly (lactic acid) and poly (butylene adipate-co-terephthalate) matrix for food packaging applications. Gum rosin acts as a plasticizer, enhancing the flexibility of the polymeric matrix, while rosin esters function as compatibilizers, boosting the toughness of the polymeric matrix (Pavon et al., 2020).

### 2.3 Oils and fats

Lipids, fats and oils contain more fatty acids and compounds related to them. Although they share a chemical structure, fats and oils differ physically, as fats are solids at room temperature while oils are liquids. They are mainly composed of mixed triglycerides that are formed by the addition of one glycerol molecule to three fatty acid molecules. A fatty acid is a chain of carbon atoms linked by hydrogen atoms, terminating in a carboxyl group (Mohamed et al., 2020). Some of the important essential fatty acids that are generally adopted in edible films and coatings are linolenic acid, oleic acid, and stearic acid (Zubair et al., 2021). These fatty acids are used as emulsifiers and dispersants to improve emulsion stability, exhibit good resistance to water vapor, and improve the mechanical properties of edible films and coatings (Nehchiri et al., 2021). Many researchers have used fatty acids as emulsifiers and dispersants to improve the functional properties of edible films and coatings (Nehchiri et al., 2021). In their work, Ma et al. (2016) incorporated oleic acid as a hydrophobic additive in tara gum edible films, resulting in decreased WVP and an improved surface contact angle (Ma et al., 2016). Similarly, Kathiresan and Lasekan (2019) investigated the addition of stearic acid to starch-based edible film, finding that stearic acid acts as an effective moisture barrier, contributing to the film's highest tensile strength (Kathiresan and Lasekan, 2019).

Fatty acids and their derivatives differ greatly in their chain length, physical properties, and saturation degree (Rhim and Shellhammer, 2005). According to Atta et al. (2022), a composite coating containing plant oils (olive oil and ginger oil) can increase the postharvest shelf life of oranges and tomatoes, prevent them from spoiling, and keep them from losing weight (Atta et al., 2022). Fangfang et al. (2019) developed potato starch-based films of virgin coconut oil with different concentrations that improved the antibacterial, mechanical, and water barrier properties (Fangfang et al., 2019). In a study by Kumar and Saini (2021), clove oil was found to be excellent at preserving the quality characteristics of tomatoes and prolonging their shelf life when used in composite coatings (Kumar and Saini, 2021).

## 3 Current strategies for improving efficacy of lipid-based food packaging

### 3.1 Techniques of coating formulation

Edible coating has been largely applied to various fruits and vegetables as protective coatings to sustain their quality for as long as possible by slowing down the process of senescence and ripening while preventing anaerobic conditions that may diminish the produce's quality. These coatings effectively manage intrinsic fruit characteristics, reducing rates of respiration and transpiration to mitigate moisture loss and enhance the visual attractiveness of produce (Ncama et al., 2018). Recent advancements in coating development have introduced various approaches, including nano-coatings, composite coatings (a mixture of hydrocolloids and lipids), and multilayer systems incorporating active agents like plant oils and fat, which may contain antioxidants and antimicrobial properties. These elements contribute to a controlled and sustainable release, influenced by the chemical characteristics and the varying degrees of interaction among their constituents (Alves et al., 2020; Magri et al., 2020; Miranda et al., 2022; Susmita Devi, Kalita, Mukherjee, & Kumar, 2022). Application methods such as spraying, dipping, panning, and brushing, followed by drying, offer flexibility and cost-effectiveness. Edible coatings not only eliminate the need for refrigeration during shipping but also facilitate the transportation of diverse produce varieties in shipping containers. Overall, the application of edible coatings stands as one of the most advanced techniques for preserving the postharvest quality of fresh produce (Ncama et al., 2018).

#### 3.1.1 Dipping method

**Fig. 1:**
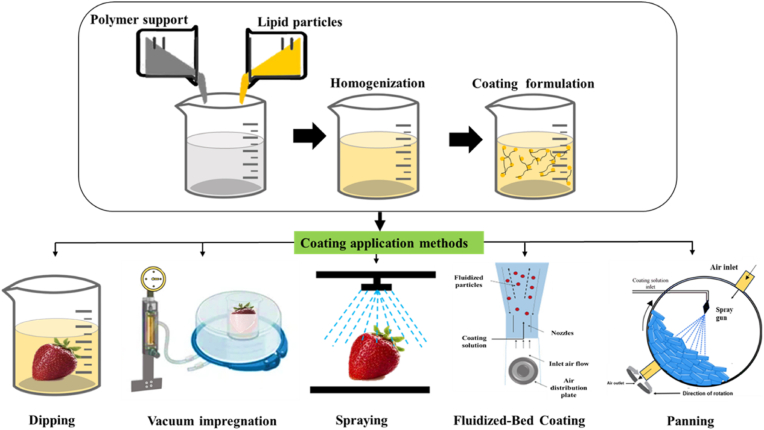
Schematic representation of different coating techniques for food applications.

Dipping is one of the most prominent coating techniques in the food packaging sector and is highly acceptable due to its easy use and affordability. In this process, a food sample is immersed in a coating-forming dispersion as showed in Fig. 1. In order to achieve the coated end products, the surfeit coating is either drained or dried. The dipping technique often only needs 0.5–5 min to thoroughly coat all food surfaces, even those with challenging or complicated surfaces (Anugrah et al., 2020). Despite its simplicity, this technique also has some limitations, including that a thick coating might cause problems with the respiration rate and storage of the food product, reduced effectiveness due to dilution and the dissolving effect, and difficulty in achieving strong adhesion of the coating solution if the food surface is hydrophilic. Usually, a layer-by-layer process is used to increase adhesion, which involves dipping food into a polyelectrolyte solution with opposite charges (Dhumal and Sarkar, 2018). The dipping method requires careful control of parameters such as temperature, viscosity, and the solvent type used in the coating solution, as well as the dipping repetition and immersion time (Tang and Yan, 2017).

#### 3.1.2 Vacuum impregnation technique

The vacuum impregnation technique is similar to the dipping method. Two airtight vacuum chamber is connected to a vacuum pump to immerse the food in a coating solution instead of using a dipping tank as presented in Fig. 1 (Anugrah et al., 2020). The principle of vacuum impregnation was linked to hydrodynamic mechanics and deformation-relaxation phenomena, where pressure drop and subsequent restoration of air pressure are the main driving forces (Zhao et al., 2021). During the vacuum application, a pressure gradient forms within the system, causing entrapped air to be drained from the intracellular spaces in the tissue of fruits and vegetables immersed in an impregnation solution under a certain vacuum pressure. Upon the restoration of atmospheric pressure, the solution can effectively penetrate the porous solid matrices due to pressure gradients. This not only preserves the natural tissue composition but also enhances texture quality and reduces drip loss. Such benefits are especially advantageous for food retention when compared to the dipping method (Lima et al., 2016). In addition, pressure changes can also cause product deformation. In certain cases, deformation can cause changes in the size and shape of pores, resulting in varying levels of impregnation. Therefore, the vacuum pressure, vacuum period, and atmospheric restoration time must all be closely monitored and controlled when using the vacuum impregnation procedure (Duarte-Correa et al., 2020). It is an innovative food processing technique with high efficiency and low cost (Nian et al., 2019).

#### 3.1.3 Spraying method

The spraying method is a common practice for applying coating solutions to food products in the form of droplets that are dispersed evenly throughout the surface of the food by an array of nozzles (Fig. 1) (Suhag et al., 2020). The droplet size of the coating depends on various factors, like the spray gun's nozzle, air flow velocity, liquid flow rate, inward air temperature, and humidity (Costa et al., 2018). The main advantages of this technique include uniform coating, thickness control, ensuring desired layers, controllable temperature, and minimizing contamination of the coating solution with the ability to manage continuous production (Andrade et al., 2012a, Andrade et al., 2012b). Multiple engineering processes and industrial applications generally employ spray systems. In the food industry, they are used in operations including washing, cooling, humidifying, packaging, and coating. The liquids' physical characteristics used in each of these applications, such as viscosity, surface tension, density, and others, may vary, and this can make the fluid behaviour unique to each liquid. As a result, various atomizers and more versatile spraying systems must be created to handle various spray applications (Tratnig et al., 2009).

#### 3.1.4 Fluidized-bed coating

Fluidized-bed coating is a method for coating food items with a very thin layer that has an incredibly low density or exceptionally small dimensions. This technique is quite flexible and can choose a variety of coating materials and the number of layers to be placed (Fig. 1). Due to the intricacy and high expense of the technology needed, this technique has not yet been widely used (Punia Bangar et al., 2021). The fluidized-bed coating technique encourages agglomeration of the powder, which improves the coating's solubility and dispersion. Hot air is used to fluidize the powder, and a binder liquid is also sprayed at the same time. This results in the attachment of particles, agglomeration, and the drying of agglomerates. Both batch and continuous processes are suitable for it (Debeaufort and Voilley, 2009).

#### 3.1.5 Panning method

The method uses a spinning pan that contains the food item to be coated (Fig. 1). To coat the food product, the coating solution is poured onto the pan, toppling the food item with a uniform base layer. The covered product is then dried using airflow or heat treatment. The technique was frequently created for medications, and it is constantly being improved for usage in the food packaging sector (Punia Bangar et al., 2021). Depending on the characteristics of the food products, this technique is divided into three kinds: hard panning, soft panning, and chocolate panning. Hard panning is the process in which sugar syrup is continuously spread over a surface until it dries and crystallises to form a hard shell. In soft panning, corn syrup and sugar are combined to make a coating that is dried by covering it with dry sugar to create a coating that is thick, soft, and contains fewer layers. While chocolate panning uses a layer of fat around a core, it is possible to use chocolate, confections made with cocoa, or a flavourful compound coating (Suhag et al., 2020).

### 3.2 Film fabrication techniques

#### 3.2.1 Casting method

**Fig. 2:**
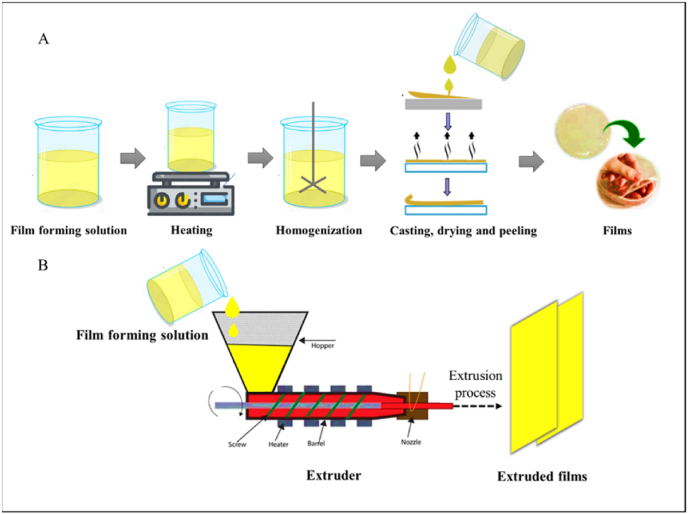
Schematic representation of different film fabrication techniques: A) Solvent casting method, B) Extrusion Methods.

Solvent casting is a traditional method of film-forming that involves evaporating solvent to increase solution viscosity, causing solid concentration to increase and intermolecular interactions to emerge (Deng et al., 2017). This method has been implemented on an industrial scale using biopolymers along with plasticizers such as glycerol, aloe, resins, etc. (Fig. 2A). After selecting the appropriate materials, the biopolymer is dissolved in the right solvent, and the solution is then cast into the molds. Degassing and drying are carried out after this phase before the film is eventually removed from the surface (Suhag et al., 2020). Casted films provide a consistent thickness distribution, good transparency, low haze, and changeable mechanical and water barrier capabilities, among other qualities (Deng et al., 2017).

#### 3.2.2 Extrusion Methods

Polymeric films are currently produced on a commercial scale using extrusion, a method of polymer processing. This method is often known as a “dry process” since it operates best with a minimal amount of water or solvents (Peressini et al., 2003). Multiple sources of biopolymers, most commonly lipids and starches, are used together with other additives in co-extrusion blowing to achieve the desired results. This method relies on the thermoplasticity of biopolymers as well as glass transition, phase transition, and gelatinization properties (Han and Aristippos, 2005). The extrusion process can be defined into three sections, such as feeding, kneading, and heating (Fig. 2B). Firstly, the mix of film components is introduced into the feeding zone and compressed with air. As a second step, pressure and temperature are increased further to further compress the materials. Lastly, heating takes place in the final section of the extruder, which experiences high temperatures, shear rates, and pressures (Rowat et al., 2021). Several factors, like screw speed, barrel temperature, die pressure, die diameter, energy input, feed moisture content, etc., are crucial for the extrusion operation in order to shape the end product (Yachuan Zhang, Rempel and Liu, 2014). As a result of the extrusion process, compact films can be formed and can withstand pressure without creating cracks or holes (Ahmed et al., 2022). While these methods are already employed in synthetic polymer production, they may be too harsh for biopolymers. However, with some adaptation, they can be utilized for creating new blends of biopolymers (Colak et al., 2016). They are also scalable for industrial applications because they produce films with superior mechanical and thermal properties in a faster and less energy-demanding manner (Silva et al., 2023). For example, protein/lipid composite films could be promising for large-scale commercial production using extrusion, which lowers the costs of protein-based materials. Additionally, these composite films improved their functional properties by combining the excellent oxygen barrier properties of proteins with the superior moisture barrier properties of lipids. According to Chevalier et al. (2018), wax powder can be incorporated into dry blend powder, and both the extrusion process and the wax source affect the properties of edible films by improving mechanical properties and hydrophobicity. Composite films could be easily produced using such a scalable industrial process, which could benefit further research in this area (Chevalier et al., 2018).

### 3.3 Composite and active films and coatings

Lipids are known for their barrier qualities and transparent nature with many beneficial properties, such as antibacterial, antioxidant, increased water vapor barrier, and other functionalities, but they have certain disadvantages like poor mechanical strength, brittleness, and a lack of homogeneity. Additionally, lipid-based films tend to be opaque and rigid, imparting a waxy and greasy texture and taste that may not be desirable for packaging materials in many cases. Hence, achieving the desired film properties necessitates compatibility between the lipid phase and the polymer matrix, which typically contains high molecular weight components (V et al., 2022). Unlike other biopolymers, lipids cannot independently form an edible film. While they can create an edible coating, the absence of numerous repeating units linked by covalent bonds prevents the formation of a standalone film. Therefore, various other lipids and hydrocolloids are incorporated into composite formulations to benefit from each other's advantages (Senturk Parreidt, Müller and Schmid, 2018). Composites can be formed as a bilayer or conglomerate to enhance the qualities of lipids and the properties of polymeric materials. For example, the stability of lipids increased in blends of free fatty acids and proteins (Chiumarelli and Hubinger, 2014). The food industry and the medical sector experienced the most significant applications of these blends and composite materials. Hence, lipids were utilized in these compositions to make films or coating materials that were more advantageous in their applications due to their great qualities. Lipids were also used as biomaterials to manufacture some polymers with lipid-like characteristics rather than blending them with lipids (Qurat ul et al., 2016). Lipid-based composites have been reported to target a wide range of applications for food packaging. In the production of biocomposites and bionanocomposites for food packaging applications, naturally occurring lipids such as waxes or oils have been employed as a matrix, filler, and plasticizer (Zubair et al., 2021).

Depending on the application, composite films can be either bilayers or stable emulsions. A bilayer composite film is formed by the deposition of lipid layers on the surface of the pre-formed hydrocolloid film. In an emulsion composite film, lipids are dispersed and entrapped in the hydrocolloid film-forming solution (Perez-Gago, Serra, Alonso, Mateos, & del Río, 2005). These composite films offer both mechanical and barrier properties by using each film's component's functional characteristics. Bilayer films provide a much higher water barrier efficiency than emulsion-based films and are often comparable to pure lipid films (Debeaufort and Voilley, 2009). However, due to the other processing steps required for filmmaking, bilayer films are not suitable for high-speed commercial production.

Cellulose ethers are the most frequently used materials for creating composite films, although starch, alginates, chitosan, pectinate, and carrageenan have all been employed to create biocomposite films (Karbowiak et al., 2007). As well as these substances, stearic and palmitic acids, beeswax, monoglycerides, and lecithin are often combined in these products (Debeaufort and Voilley, 2009). The development of edible films from lipid molecules has been the subject of several studies. In a study, a wheat gluten-based edible film was developed by incorporating lipids, and the effects of lipids were examined for their barrier, mechanical, and physical properties. The results showed that water affinity and WVP both decreased. The film's surface hydrophilicity with the addition of lipid was lower. Though the addition of lipids resulted in weak structures within the film matrix due to reduced bond formation, a physical change occurred between the glass and rubber-like states of films when lipids were added (Rocca-Smith et al., 2016). In another study, b-glucan fatty acid esters were added to the arabinoxylan-based films to analyse various properties. As a result, it was demonstrated that the addition of b-glucan-fatty acid esters enhanced the thermal stability of the films as compared to the controls (arabinoxylan-alone and arabinoxylan-b-glucan films) (Ali et al., 2017).

## 4 Effects of lipid on biopolymer-based films and coatings

### 4.1 Improved thermal, and mechanical packaging systems

In the food packaging sector, mechanical strength and thermal characteristics are important for the protection and commercialization of foods. The mechanical properties of materials refer to their ability to exhibit certain properties when forces and heat are applied to them. Lipids and their derivatives are discussed in relation to their effects on the thermal and mechanical properties of food packaging materials. In general, lipids improve the barrier, but their concentration and nature may affect the mechanical performance of films or coatings. Therefore, lipid constituents need to be optimized depending on the application since excessive addition and non-compatibility with the matrix may lead to stiff, weak, and brittle films, resulting in reduced elongation and elastic modulus as well as tensile strength (Zubair et al., 2021). According to Zahedi et al. (2010), the addition of fatty acid affected the mechanical properties of pistachio globulin protein-based films; the elongation at break was reduced significantly (35–70%), and the ultimate tensile strength was also found to be declining (Zahedi et al., 2010). On the other hand, Wang et al. (2016) stated that the incorporation of epoxy castor oil into soy protein-based films improved the films mechanical properties by nearly a factor of 2, and elongation at break increased by about 23%. The hydrogen bonding between long chains of epoxidized castor oil and soy protein isolates was attributed to this improvement in the material's structural analysis (Wang et al., 2016). However, after a comprehensive review of the literature, it was determined that there is limited information on the impact of using lipids to improve the mechanical properties of various biopolymers for food packaging. However, it was observed that incorporating hydrophilic materials like proteins, starch, chitosan, and cellulose into the lipid materials can enhance both the mechanical and thermal properties (Zubair et al., 2021). Recently, ultrasound technique has been increasingly used in film making to improve polymer film properties. Liu et al. (2018) studied the ultrasound effect on starch/lauric acid-based films. This study improved the films' mechanical performance. At ultrasonic amplitudes up to 40%, there was more complexation between lauric acid and starch. This can be attributed to improved lauric acid dispersion and amylose release due to starch grain disintegration. The tensile strength was increased from 6.2 to 8.31 MPa; however, a further increase in ultrasonic amplitude (70%) disrupted complex formation and reduced tensile strength (Liu et al., 2018). A study by Kang et al. (2020) also showed that ultrasonication improves the mechanical properties of starch-lipid composite films (Kang et al., 2020).

### 4.2 Improved barrier properties; water vapor permeability

The barrier properties of food packaging film play an important role in predicting or estimating the shelf life of food products. A major function of food packaging film is to retard the transfer of molecules between food and the environment, to preserve food quality. These properties can be measured to determine the permeability and transfer of molecules such as water vapor, organic vapor, or gaseous gases through a film. Among these, WVP is extensively studied in biodegradable films due to its significant impact on food freshness, crispiness, or dehydration prevention, depending on the food type (Cazón and Vázquez, 2020). The hydrophobic and crystalline nature of lipid-based materials have a direct impact on their barrier properties, which vary with the degree of unsaturation, aliphatic chain length, and the type of chemical functionality. Film structure also affects barrier properties, depending on the film preparation technique used. As a result of the penetrant molecule's properties, it also affects the interaction with the film components and the permeability of the film (Zubair et al., 2021). Lipid-based materials have extremely low WVP values, making them effective moisture barriers because of their nonpolar characteristics (Tyagi et al., 2021). Lipids such as waxes, oils, and fatty acids are commonly used in film-forming solutions. However, the brittle nature of lipids can cause adherence and homogeneity when used alone, leading to their frequent combination with hydrophilic hydrocolloids in emulsion or bilayer forms (Khodaei et al., 2021). A variety of hydrocolloids have been used to form composite films, including cellulose, chitosan, pectin, starch, alginates, and carrageenan. Wang et al. (2014) stated that the WVP of pistachio globulin protein films incorporated with fatty acids decreased exponentially with an increase in the concentration of fatty acids due to increased hydrophobicity. The WVP was decreased by about 3% compared to films without fatty acid (Wang et al., 2014). Several authors have observed consistent results regarding the incorporation of lipids such as stearic acid into rice starch-ι-carrageenan edible films (Thakur et al., 2016), oleic acid into wheat bran cellulose/wheat gluten composite films (Shen et al., 2022), and palmitic or caproic acid into the fish proteins films (Pereira et al., 2020) tend to show reduced WVP and solubility of the food packaging films. On the other hand, waxes were shown to be among the most effective when the components' hydrophobicity increased, which resulted in a decrease in the WVP. Biopolymer films with added waxes such as candelilla wax (Kowalczyk and Baraniak, 2014), beeswax (Wardhono et al., 2019), and carnauba wax (de Oliveira Filho et al., 2021) have been reported to have excellent moisture barriers for sodium caseinate-based films, starch-based gluten films, and chitosan-based films, which is one of the most important characteristics of a good food packaging material. Similarly, Nurul Syahida et al. (2020) also studied the application of fish gelatin-based films by incorporating palm wax and its effect on the water barrier properties of food packaging. It was found that the addition of 15% palm wax significantly (p < 0.05) reduced the WVP of the films. As the hydrophobic wax binds to the gelatin network, hydrogen bonds between water molecules are inhibited, thereby reducing water adsorption (Nurul Syahida et al., 2020). The emulsified films have the greatest influence on the water vapor barrier based on the type of lipid and the relative polarity of the support polymer (Wang et al., 2014). It is important to note that the barrier property of biopolymer films is also influenced by pinholes or cracks in the film that may facilitate water vapor diffusion (Kowalczyk and Baraniak, 2014).

### 4.3 Antioxidant activities

Packaging materials with antioxidant properties are being developed by incorporating active compounds into the polymer matrix; however, the carrier itself may also possess antioxidative properties (Kowalczyk and Baraniak, 2014). The effectiveness of films and coatings to preserve fruit and vegetable quality after harvesting is due to their antioxidant and antibacterial capabilities (Susmita Devi et al., 2022). In active food packaging, natural antioxidants are preferably incorporated into biopolymers to prevent off-Flavors and undesirable textures, thereby increasing shelf life. A few examples of lipid-based antioxidants used in food packaging include essential oils, phospholipids, phenolipids, and lipophenols. A phenolic compound and flavonoid present in these components contribute to their antioxidant properties (Zubair et al., 2021). Pires et al. (2013) reported that the antioxidant activity of hake protein films generally increases with the inclusion of essential oils, as evidenced by DPPH radical scavenging activity and reducing power (Pires et al., 2013). Similarly, several other studies found that sodium caseinate-derived films containing ginger and cinnamon essential oils and clove essential oil incorporation into sunflower protein concentrate-based films at varied concentrations have good antioxidant properties (Salgado et al., 2013). In addition, waxes like beeswax have antioxidant and antimicrobial activities, wound healing, anti-stress, and anti-inflammatory properties, and carnauba wax showed antioxidant and antiprotozoal properties (Shakeri et al., 2019). The availability of gallic acid, chlorogenic acid, and catechin in aqueous extracts of carnauba wax types A and B was also proven by HPLC analysis. These findings suggest that carnauba wax has antioxidant activity that is beneficial for pharmaceutical and food industry uses, particularly food packaging (Susmita Devi et al., 2022).

### 4.4 Antimicrobial properties

Antimicrobial food packaging has piqued scientific and technological interest in recent years. Antimicrobial systems, or agents, are active packaging in which the combined activities of the packaging material, product, and atmosphere inhibit the growth of bacteria in the food. The most common food packaging pathogens include *Bacillus cereus, Listeria monocytogenes, Escherichia coli O157, Aspergillus niger, Saccharomyces cerevisiae, Campylobacter, Clostridium perfringens, and Staphylococcus aureus*. An ideal food packaging should not only protect the food from contaminants and food-borne diseases but also maintain its structural stability (Duda-Chodak et al., 2023). Researchers frequently test the antimicrobial activity of developed packaging systems in vitro before shelf-life assessment. Different approaches used in developing antimicrobial packaging systems include physically mixing microbial agents into a polymer matrix during processing and covalently attaching the active agent to a macromolecule utilized in packaging material manufacture (Dini, 2016).

Edible films and coatings are treated with a variety of antimicrobial agents. These antimicrobial agents are activated in food in three ways: by releasing the agent, immobilizing it, and absorption system during storage, thereby enhancing food safety (Abdollahzadeh et al., 2021). According to Akyuz et al. (2018), various plant oils and animal fats like corn, sunflower oil, olive, and butter were investigated for their impact on the environmental properties of chitosan packaging materials. A further study examined how the degree of unsaturation of fats and oils effects on film properties. It was found that olive oil/chitosan films had the highest antimicrobial properties of all films, comparable to the commercial antibiotic gentamicin (Akyuz et al., 2018). Waxes such as carnauba wax, beeswax, or candelilla wax have been used in the development of bioactive coatings.

Gonçalves et al. (2010) demonstrated effective postharvest preservation of plums and nectarines treated with 4.5% and 9% carnauba wax against molds like *Monilinia fructicola* and *Rhizopus stolonifera*, which cause rot in plums and nectarines, respectively (Gonçalves et al., 2010). Saucedo-Pompa et al. (2009) reported that the candelilla wax-based edible film significantly reduced the damage caused by *Colletotrichum gloeosporioides* on avocados (Saucedo-Pompa et al., 2009). Using beeswax nanoparticles, Shakeri et al. (2019) improved the biological activities of antimicrobial compounds, namely astaxanthin and carvacrol. By entrapping carvacrol and astaxanthin in beeswax nanoparticles, more than 80% of total *Staphylococcus aureus* and *Pseudomonas aeruginosa* biofilm cells were killed (Shakeri et al., 2019).

### 4.5 Hydrophobicity

Lipids are safe, edible, hydrophobic, natural substances comprised of waxes, resins, fats, sterols, fat-soluble vitamins, and so on. They are among the major sources of energy and have a crucial role in the functions of the cell and membrane. Lipid hydrophobic nature and their structure, have a direct influence on the barrier, which differs depending on the degree of unsaturation, aliphatic chain length, and the type of chemical functionality (Yousuf et al., 2022). Lipid hydrophobicity allows them to form stable structures such as lipid droplets or lipoproteins, which can be employed to store and transport energy-rich compounds such as triglycerides or cholesterol. Natural waxes, such as rice bran wax, candelilla wax, carnauba wax, and beeswax; vegetable oils; acetoglycerides and fatty acids; and resins, like shellac and wood rosin, are hydrophobic substances that are successfully applied to lipid-based edible films and coatings (Rhim and Shellhammer, 2005). As a result of their low polarity, lipids are alleged to be extremely effective at preventing moisture transfer between food and packaging. The WVP of the material decreases as the hydrophobic (lipid) phase concentration increases. Among lipids, waxes showed better barrier properties due to the presence of excellent hydrophobic components (Zubair et al., 2021).

An edible super-hydrophobic coating was developed from beeswax and coffee and analysed for hydrophilicity via contact angle measurements. After continued heating and flushing, the apparent contact angle of this coating may stay above 150° (hydrophobic). This thermo-resistant edible super-hydrophobic coating is a potential solution to the problem of non-resistance to high temperatures in conventional edible super-hydrophobic coatings and has many possible applications in the field of functional food packaging (Zhang et al., 2019). Similarly, the incorporation of oils decreased the WVP of bio-based food packaging materials. The hydrophobic properties of oils enhance the hydrophobic phase of the material, thereby minimizing water diffusion through films or coatings (Zubair et al., 2021).

## 5 Lipid as a nanoparticle in the development of edible films and coatings

Lipid nanoparticles consist of a lipid matrix, emulsifiers, co-emulsifiers, water, and bioactive compounds. They are designed for transporting bioactive compounds and adhere to principles governing compound delivery and release in the body. In food processing, these carrier systems can safeguard desired compounds from degradation, preventing unfavourable chemical reactions and loss of functional activity due to exposure to light and oxygen (da Silva Santos, Badan Ribeiro and Andrade Santana, 2019). A nanoscale material with diverse physicochemical properties can improve the functionality of food packaging materials, which can address challenges associated with macro- and microstructure systems, such as compatibility with the food matrix resulting from aggregation and phase separation (Zubair et al., 2021). Since early times, various lipid-based materials have been utilized in the formation of edible food packaging due to their effectiveness in reducing WVP, thereby influencing the properties of edible films and coatings. In contrast to traditional lipid materials, lipid nanoparticles have emerged as an alternative system to emulsions, liposomes, and polymeric structures. These nanoparticles address many shortcomings associated with the unavailability of biocompatible polymers and emulsifiers, as well as the use of organic solvents not approved for food systems. Moreover, the integration of lipid nanoparticles in edible food packaging demonstrates improved effectiveness in safeguarding food products from environmental damage (Ghosh et al., 2021). Lipid nanoparticles are presently classified as solid lipid nanoparticles (SLNs) and nanostructured lipid carriers (NLCs). SLNs are effective colloidal carriers with particle sizes ranging between 50 and 1000 nm and a lipid core that remains solid at both body and room temperatures. This characteristic enables high entrapment of hydrophobic drugs with a controlled release profile. SLNs can be enhanced by incorporating liquid lipids into the solid structure, resulting in NLCs, which offer improved loading capacity and greater compositional stability (Abdel Samie and Nasr, 2020). Recently, Shakeri et al. (2019) developed SLNs using beeswax to enhance the biological activities of antimicrobial agents like astaxanthin and carvacrol. Their study revealed that encapsulating carvacrol and astaxanthin within beeswax SLNs resulted in increased radical scavenging ability and effectively eradicated over 80% of total *Pseudomonas aeruginosa* and *Staphylococcus aureus* biofilm cells. This enhancement is attributed to the improved water solubility and dispersibility of astaxanthin and carvacrol within the SLNs (Shakeri et al., 2019). Similarly, Zambrano-Zaragoza et al. (2020) investigated the impact of an edible coating based on beeswax SLNs on the preservation of strawberries. Their findings revealed that coating strawberries with beeswax SLNs (10 g/L) resulted in the lowest weight loss (6.1%), a decay index of 31%, and a loss of firmness of 34%. This approach proved to be an excellent alternative for the conservation and extension of the shelf life of strawberries stored under refrigeration (Zambrano-Zaragoza et al., 2020). Using lipid nanoparticles in the development of edible films and coatings has several advantages, including reducing senescence, reducing the natural maturation rate, and imparting antimicrobial and antioxidant properties to food products through the components of the coating materials.

## 6 Applications of lipid-incorporated biopolymer-based food packaging materials

### 6.1 Films for packaging of fruits and vegetable

Fresh produce has a short shelf life and deteriorates quickly because of its perishability. Poor preharvest and postharvest handling of commodities, as well as inadequate marketing and processing infrastructure, contribute to higher losses. Post-storage techniques are needed to maintain a commodity's nutritional value and freshness. Various storage techniques require a low temperature to preserve commodities, but their high cost and shortage of supply in different areas limit their use (Hasan et al., 2019). Biopolymer-based films can be used as a sustainable alternative to food packaging. However, biopolymers derived from plants, animals, and microorganisms lack some basic physicochemical and mechanical properties. By reinforcing biopolymers with nanoparticles, functional qualities such as antibacterial and/or antioxidant activities can be added to composites to solve these inadequacies (Basumatary et al., 2022). Lipid functionalization enhances the ability of colloidal emulsions and suspension phases to form films, facilitating the adherence of those films to food surfaces. Also, lipids serve many functions, including emulsifiers, texturing agents, antioxidants, antimicrobial agents, enzymes, and process aids (Liyanapathiranage et al., 2023). Lipid-based films have a low polarity, which makes them good at preventing moisture penetration. Fruit and vegetables benefit from moisture control, which is essential to extending shelf-life post-harvest. Moreover, lipids preserve the texture and flavour of food by acting as carriers for active molecules (Hassan et al., 2018). Incorporating lipids with hydrocolloids like polysaccharides, proteins, cellulose, starches, and their derivatives to create composites, nanocomposites, and cross-food packaging films enhances antimicrobial efficacy as well as improves thermal, mechanical, barrier, and optical properties (Abdul Khalil et al., 2018; Oliveira Filho et al., 2020).

**Fig. 3:**
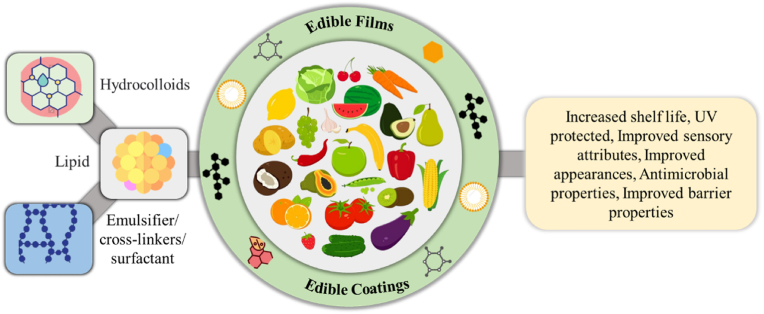
Schematic illustration of biopolymeric films and coatings incorporating lipid for fruits and vegetables packaging during storage.

For example, Karimi Khorrami et al. (2021) developed alginate-based films by integrating palm oil. This addition notably enhanced the UV-absorbing capabilities of the films without substantially affecting their transparent appearance. With increasing palm oil concentration, the films' tensile strength, elastic modulus, and swelling ratio decreased, while their thermal stability, WVP, and contact angle increased. This study highlights the successful incorporation of lipid into calcium-alginate films, enabling modulation of their physicochemical attributes (Karimi Khorrami, Radi, Amiri and McClements, 2021). Similarly, Nurul Syahida et al. (2020) studied the effects of palm wax on fish gelatin films. The findings revealed that incorporating palm wax into the film enhanced tensile strength while decreasing solubility and swelling compared to films without palm wax, showing a great potential for food packaging applications (Nurul Syahida et al., 2020). Oliveira et al. (2018) also developed a biofilm hydrophobized with 10% beeswax that showed a beneficial effect on water vapor transmission with an 80% rise in elasticity and a 15% decline in solubility. The filmogenic solutions used as a coating improved the physical-chemical analysis of guavas by reducing weight loss, ensuring adequate ripening during 15 days of storage under a controlled environment (15 °C ± 2 °C, 90% ± 2% RH), and showing better acceptability with improved sensorial attributes of the fruits (Oliveira et al., 2018). Fig. 3 shows a schematic image of biopolymeric films and coatings incorporating lipid for fruits and vegetables packaging during storage. The study shows that lipid-based materials are employed to produce edible films. The relevant works are discussed in Table 1.

**Table 1:** Use of lipid components incorporated into edible composite films to improve their functioning.

Lipid component	Type of composite film	Effects on composite film due to lipid component	References
Rapeseed oil (1 & 3 % w/v)	Whey protein isolate film	The incorporation of lipid enhanced the hydrophilicity, reducing moisture content and water solubility of the films.	Galus and Kadzińska (2016a)
Rapeseed oil (18 % w/v), beeswax (18% w/v)	Starch/gelatin edible films	The incorporation of lipid improved the surface hydrophobicity, mechanical strength, water vapor barrier and water resistance properties indicating the formation of a strong film network structure.	Cheng et al. (2021)
Beeswax (10–20% w/v)	Agar/maltodextrin films	The incorporation of lipid improved the films hydrophobicity. Film with 10% beeswax achieved the lowest WVP (6.86 × 10^−13^ g/m^−1^ s^−1^ Pa^−1^) and oxygen permeability (1.17 × 10^−17^ cm^2^ s^−1^Pa^−1^), respectively. Additionally, the tensile strength, elongation at break, and Young's Modulus of the films increased with the presence of lipid, indicating promising potential for food packaging.	(Yiwen Zhang et al., 2019)
Carnauba wax (15 wt %), *Mentha spicata* or *Cymbopogon martinii* essential oil (0.1%, 0.2%, & 0.3% v/v)	Arrowroot starch-based films	The incorporation of lipid reduced the moisture content and WVP, improved thermal stability of the film and provided excellent protection against fruit-spoiling growth, and ultraviolet and visible light.	de Oliveira Filho et al. (2021)
Almond or walnut oils (0.5 and 1.0% w/v)	Whey protein edible films	The incorporation of oils leads to opaque films with heterogeneous microstructure and reduced swelling, water vapor permeability, and surface hydrophilicity of the films. Also, increase oxygen and carbon dioxide permeability with increased in oil content. Almond oil had a stronger plasticizing effect and was more effective in modifying the properties of films.	Galus and Kadzińska (2016b)
Carnauba wax (0.5, 1.0, & 1.5%)	Sodium alginate and whey protein-based films	The incorporation of wax at a higher concentration (1.5%) enhanced the moisture barrier properties and reduced the tensile strength (3.92–3.34 MPa). At lower concentrations (0.5%) films showed uniform microstructure and better transparency but lack in moisture barrier properties.	Butt et al. (2023)
Candelilla or carnauba wax (0.5 or 1.0% w/w)	Sodium caseinate films	The incorporation of waxes increased the tensile strength from 1.91 to 2.60 MPa compared to the control film, indicating that the addition of wax resulted in more resistant films. However, it also led to increased total colour differences, film opacity, and a decrease in WVP, elongation at break, and film lightness.	Galus et al. (2020)
Olive oil (10% w/v), Chitosan (2%, wt./v)	Chitosan based film with cellulose nanocrystals	The incorporation of lipid effective reduced the inherent high WVP and total soluble matter. Additionally, the tensile modulus was significantly increased through the addition of cellulose nanocrystals, while the use of olive oil moderated the reduction in elongation at break. This synergistic effect represents an improvement beyond the anticipated response.	Pereda et al. (2014)
Stearic acid (0.3–0.9%, w/w)	Starch carrageenan edible film	The incorporation of lipid at a lower concentration resulted in films with minimized WVP values, reduced thickness, improved solubility, and enhanced mechanical properties. This led to films with superior physical, and barrier overall.	Thakur et al. (2016)
Mixture of acetic esters of mono- and diglycerides and beeswax (25 wt %)	Wheat gluten based edible films	The incorporation of lipid decreases the water sorption, water affinity (hydrophilicity), and water transfer of the films. Additionally, mechanical properties are influenced by the lipid addition, resulting in decreased rigidity and, at high water activity, an increase in extensibility.	Rocca-Smith et al. (2016)
Candelilla wax (0, 5, or 7.5% w/v)	Whey protein isolate-based films	The incorporation of lipid had a impact on barrier properties with increasing wax concentration. As the concentration of the wax increased, the oxygen permeability increased while WVP decreased. Additionally, the tensile strength of the film decreased, while the elastic modulus and percentage elongation at break did not change significantly. However, the films exhibited increased strength and flexibility, resulting in films with better overall properties.	Janjarasskul et al. (2014)
Shellac (20–60% w/v), stearic acid (0–2% w/v)	Pea starch-guar gum films	The incorporation of lipid improved the mechanical properties of the film, with a reduction in tensile strength and increased in elongation at break attributed to the addition of fatty acids. Meanwhile, provided the film with minimum thickness and WVP.	Saberi et al. (2017)
Shellac (10% w/w)	Konjac glucomannan/shellac film	Incorporating shellac enhanced the thermal tolerance and mechanical properties of the films, including tensile strength and elongation at break. Moreover, the presence of lipid in the films resulted in improved water resistance, making them promising materials for food packaging applications.	Du et al. (2019)
Beeswax (25% & 50% w/w)	Chitosan nanoparticle matrix with cellulose nanocrystals films	Incorporating beeswax improved the homogeneity and stability of the film-forming emulsion, thereby enhancing the water vapor barrier of the film. However, it also led to a deterioration in the strength of the film.	Wardhono et al. (2019)

### 6.2 Coatings for the shelf-life extension of fruits and vegetables

Edible coatings have shown a substantial effect on maintaining the postharvest quality of many fresh fruits and vegetables. It provides a promising result for controlling the physiological effects and physicochemical properties (Maringgal et al., 2020). Therefore, natural polymer-based novel coatings could resolve conventional food preservation shortcomings. Biopolymer-based coatings have been proven to be an excellent sustainable approach due to their intrinsic useful features like antioxidant, antibacterial, anti-browning, flavouring, etc. (Gupta et al., 2022). The type of biopolymer used determines the effectiveness of any edible coating. In the past few years, many studies have reported that lipid alone or lipid-based composite coating has been successfully characterized as an advantageous option for prolonging the postharvest shelf-life of fruits and vegetables (Khorram et al., 2017; Milani and Nemati, 2022; Susmita Devi et al., 2022). Lipid coating also reduces scratches and damages during handling and processing. Coatings such as shellac and wax are often used to improve fruit and vegetable gloss (Khorram et al., 2017; Milani and Nemati, 2022). In a study conducted by Gutiérrez-Pacheco et al. (2020), carnauba wax was added to chitosan-based coatings containing oregano essential oil to mitigate water loss and microbial spoilage in fresh cucumbers, resulting in significantly less moisture loss and effectively delayed microbial spoilage when compared to the pristine chitosan-coated cucumbers (Gutiérrez-Pacheco et al., 2020). Candelilla wax-based coatings applied to fresh strawberries infused with *Bacillus subtilis* showed a considerable potential to decrease the occurrence of deterioration in strawberries. This was achieved by decreasing the decay by almost 100% compared to the uncoated on day 6 (Oregel-Zamudio et al., 2017). Martínez-Romero et al. (2019) also study the effect of rosehip oil coating on the postharvest quality parameters and ripening process of plums. According to the findings, the developed coating significantly reduced the ethylene production and respiration rate in coated plums stored at 20 °C or at 2 °C. The fruit quality parameters, including titratable acidity, total soluble solid, total soluble solid/titratable acidity ratio, firmness, and colour, decreased and the antioxidant properties increased due to hydrophilic and lipophilic compounds present in the formulation (Martínez-Romero et al., 2019). Table 2 shows some of the applications for adding lipids to biopolymeric matrixes to extend fruits and vegetables shelf life.

**Table 2:** Lipid-incorporated biopolymer coatings applied to various fruits and vegetables.

Lipid component	Biopolymer composition	Fruits and vegetables	Coating techniques	Effects on produce	References
Beeswax (10 % w/v)	Hydroxypropyl methylcellulose 5% (w/v)	Red guavas	Dipping	The developed coating solution created a modified atmosphere around the fruit, thereby delaying the ripening process, reducing mass loss, preserving the green colour, and enhancing firmness compared to the control fruit. This treatment extended the conservation time of the guavas by at least six days, thereby demonstrating the most effective results in maintaining fruit quality.	Formiga et al. (2019)
Candelilla wax (0.15%–0.25% w/v)	Whey protein (2%–3% *w*/*v*), *Flourensia cernua* extract (0.05% w/v) and glycerol (2%, 2.5%, and 3% v/v)	Tomato	Dipping	The developed coating formulation reduced weight loss and firmness and extends the shelf-life of up to 10 days at ambient temperature.	Ruiz-Martínez et al. (2020)
Candelilla wax (0.16% w/v)	Polyphenol Larrea; PLE extract (320–920 ppm), Pectin 1.1% (w/v), aloe mucilage (5% w/v), and glycerol (0.3% v/v),	Avocado	Dipping	The developed coating formulation with higher concentration of PLE shows the most effective antibacterial activity to protect and prevent endocarp damage from fungus invasion during storage.	Aguirre-Joya et al. (2019)
Carnauba wax (10 % w/v)	Sodium dodecyl sulfate (2% w/v), Sodium alginate (0.5% w/v), poly ethylene glycol (2.5% w/v)	Eggplant	Dipping	The developed coating solution improved the commercial attributes like glossiness, early drying nature, reduced weight loss, firmness during storage and increase shelf life of up to 12 days at ambient temperature.	Singh et al. (2016)
Rosehip oil (2% v/v)	–	'President’ and ‘Royal Rosa’ plums	Dipping	The developed coating formulation significantly reduced the ethylene production and respiration rate in coated plums during storage while maintaining the plum quality properties for longer periods compared to control fruit.	Martínez-Romero et al. (2019)
Carnauba wax (17.5% w/v), oleic acid (2.8% w/v), and myristic acid (0.7 g)	Glycerol monolaurate (0.5% w/v)	Sweet potato roots	Dipping	The developed coating formulation successfully minimized weight loss and respiration rate while inhibiting decay incidence compared to the control during storage. Additionally, the coated roots exhibited stronger inhibition of root rot than the control, preserving food quality and extending the shelf life of sweet potato roots.	Aayush et al. (2022)
Carnauba-shellac wax and, lemongrass oil (0.3% v/v)	–	‘Fuji’ apples	Dipping	The developed coating formulation containing lemon grass oil reduced the total aerobic bacteria count, as well as yeast and molds, improved sensory values, minimized quality loss and extend the shelf life for up to 5 months.	Jo et al. (2014)
Beeswax (0, 10, 20, and 3 % w/v)	Xanthan gum (0.3 % w/v) and propylene glycol (0.5% w/v)	Strawberry	Dipping	The developed coating formulation effectively reduced decay rates, as evidenced by decreased fungal growth, weight loss, and physiological damage by the end of storage, thereby making a positive contribution to preservation efforts.	Zambrano-Zaragoza et al. (2020)
Beeswax (5%, 10% w/v)	cassava starch (2% w/v), corn starch (3% w/v), and gelatin (5% w/v), saponified sunflower oil (5% v/v)	Guava	Dipping	The developed coatings formulation preserved the fruits quality with good physicochemical and organoleptic properties for a longer duration by delaying mass and chlorophyll loss during storage.	Oliveira et al. (2018)
Beeswax (0.5% w/v)	Chitosan (1% w/v), glycerol (0.2% w/v), pollen grains (0.5% w/v)	‘Le Conte’ pears	Dipping	The developed coatings formulation significantly reduced the weight loss, decay index and softening rate during storage.	Sultan et al. (2021)
Beeswax (0.5%, 1 % w/v)	Starch (2% w/v)	Blackberries	Spraying	The developed coating formulation exhibits different barrier properties that did not obstruct the blackberries stomata, preserved fruit cuticle integrity, and maintained hardness.	Pérez-Gallardo et al. (2015)
Beeswax (0.8% w/v), carnauba wax (0.8% w/v), glycerol monostearate	Hydroxypropyl methylcellulose (2% w/w)	‘Mollar De Elche’ Pomegranates	Dipping	The developed coating formulation emerged as the most promising treatment, demonstrating reduced weight loss and decay while preserving the physicochemical and sensory quality of the pomegranates without any adverse effects.	Di Millo et al. (2021)
Rice bran wax (5%, 10% and 15% w/v)	–	Tomato	Dipping	The developed coating formulation enhanced the shelf-stability of tomatoes, and the 10% (w/v) wax coating showed effective control over spoilage, preserving the tomatoes for up to 27 days.	Abhirami et al. (2020)
Shellac (3.75–4.0 % w/v)	Shellac (3.75–4.0 % w/v), aloe gel (1.1–1.2 % w/v)	Tomato	Dipping	The developed coatings formulation delayed senescence shows restricted changes in ethylene synthesis and respiration rates, firmness and the prolong the shelf-life up to 12 days under ambient conditions (28 ± 2 °C).	Trinh et al. (2022)
Shellac (5 % w/v)	Sodium alginate (0.5 %), PVA (3 % w/v),	Green chillies	Dipping followed by modified atmosphere packaging	The developed treatments reduced the metabolic activities, preserved the quality attributes during storage and extend the shelf-life for up to 48 days at low temperatures (8 ± 1 °C)	Chitravathi et al. (2016)
Shellac (6% w/w)	Gelatin (10%–50% w/w)	Banana	Dipping	The developed coatings formulation successfully reduced weight loss, delayed the ripening process, total molds/yeasts count and increase their postharvest shelf life.	Soradech et al. (2017)
Shellac (5% w/v)	Starch (0.25% w/v), sodium alginate (0.5% w/v), EDTA (0.1% w/v)	Green chillies	Dipping	The developed coatings formulation was preserved the quality of fresh chillies and extend the shelf-life for up to 12 days with a higher retention of ascorbic acid, firmness and chlorophyll content during ambient storage.	Chitravathi et al. (2014)
Shellac (9%–11% w/v)	–	‘Valencia’ oranges	Dipping	The developed coatings formulation maintained the postharvest quality giving the best fruit gloss and appearance without reducing any flavour as well as retain firmness and reduced weight loss.	Khorram et al. (2017)

## 7 Safety prospect of a lipid-based edible coating

Biocompatible lipids are generally recognised as safe (GRAS) for human consumption by the Food and Drug Administration (FDA) and are used in the production of lipid nanoparticles in combination with surfactant(s), solvents, and medicinal compounds. Lipids such as waxes, fatty acids, fatty alcohols, triglycerides, and butter have been used in the production of lipid nanocarriers (Chauhan and Singh, 2022). The absence of wax will be detrimental to the delivery of products over long distances. Yet, some customers have voiced concerns regarding their use. Concerns may be raised about the provenance of its vegetarian or non-vegetarian animal-based waxes, such as oleic acid, or pesticide entrapment. As per the European directives, CODEX, and FDA regulations, edible coatings are defined as coatings made from food origin items, additives, ingredients, packaging materials, or contact substances. All film formation components and additions should be non-toxic and food-grade (Panghal et al., 2018). Regulatory bodies like the European Union and the Codex Alimentarius have authorized the use of lipid compounds like beeswax (INS 901/E 901), candelilla wax (INS 902/E 902), carnauba wax (INS 903/E 903), and shellac (INS 904/E 904) as food additive and ingredients according to good manufacturing practices (GMP) (Alimentarius, 2023).

As per the FAO/WHO food standards, it is legal to use it as a coating agent for the surface treatment of fresh fruits and vegetables, or as an additive having features such as anticaking agent, carrier, acidity regulator, glazing agent, thickening agent, surface finishing agent, and release agent in toppings, chewing gum, sauces, processed fruits, soft candy, or bakery products (Alimentarius, 2023). The European Union has legalized the use of waxes for surface treatment, having a maximum permitted level (MPL) of 200 mg/kg for carnauba wax ("Scientific Opinion on the re-evaluation of carnauba wax (E 903) as a food additive,” 2012) and candelilla wax (Aranda-Ledesma et al., 2022) and 500 mg/kg for beeswax (El Agrebi et al., 2021). In Europe and the United States, other lipid materials like palm oil and corn oil are included as vegetable oils and oleic acid (E 570) is used as a coating and emulsifying agent as per the U.S. Code of Federal Regulations and the European Union (Directive 95/2/EC) (Pérez-Gago and Rhim, 2014). The use of potentially harmful post-harvest treatments must be closely monitored by the pertinent health and food safety authorities. There should be a provision for legal proceedings if the act's requirements are violated (Panghal et al., 2018).

## 8 Conclusion and future perspectives

Edible films and coatings have been shown to increase food quality and shelf life by controlling moisture, oxygen, lipids, aroma, and flavor compounds in food systems while reducing the use of synthetic plastic packaging as they are naturally occurring, low-cost, and renewable. Various biopolymer materials, including polysaccharides, proteins, lipids, and their combinations, have been utilized in the production of films and coatings. Lipid materials like edible fatty acids or waxes are incorporated into the hydrophilic biopolymer to prepare edible films with flexibility, hydrophobicity, and cohesion. Edible coatings composed of lipids act as effective barriers against moisture and, to some degree, oxygen, thus preserving food quality. Lipid-based coatings are used for numerous applications, including food, agricultural, and pharmaceutical applications. On the other hand, lipid coatings quickly oxidize and provide a lipid flavour that detracts from the food's sensory qualities and appeal. Since some lipids are solid at ambient temperature, applying them can be challenging if solvents or high temperatures are required. As a result, in-depth research and development activities are required to resolve these issues regarding the coatings in order to make them desirable and practical and to maximize their utility to mankind. Also, the application of lipid coatings and films for novel food products or unconventional substrates, such as ready-to-eat food, meat alternatives, or dairy products, remains highly limited. However, advancements in research and innovation in barrier technology for extending the shelf-life of foods could yield significant benefits. There is a need for further study and characterization of lipid coatings and films as potential edible packaging by researchers. Composite coatings and films may also be developed to combine the advantages of lipids and hydrocolloid constituents. The effectiveness of edible coatings and films relies on their components' properties. Thus, it's crucial to use the right composition and proportions to enhance food quality and shelf life. Implementing new technologies for better control over the characteristics and functionality of coatings or films is thus imperative. Furthermore, there remains a paucity of research on the influence of lipids on the properties of edible films. Therefore, future research efforts should focus on examining the significant properties of biodegradable edible films containing various lipid substances. Additionally, other mechanisms for interaction should be explored and applied to food systems to assess their potential for improving quality and prolonging the shelf-life of foods.

## CRediT authorship contribution statement

**L. Susmita Devi:** Conceptualization, Data curation, Investigation, Methodology, Writing – original draft, Writing – review & editing. **Amit K. Jaiswal:** Conceptualization, Investigation, Supervision, Validation, Writing – review & editing. **Swarna Jaiswal:** Conceptualization, Investigation, Supervision, Validation, Writing – review & editing, and.

## Declaration of competing interest

The authors declare that they have no known competing financial interests or personal relationships that could have appeared to influence the work reported in this paper.

## Data Availability

No data was used for the research described in the article.
